# Interactions of Cortisol and Prolactin with Other Selected Menstrual Cycle Hormones Affecting the Chances of Conception in Infertile Women

**DOI:** 10.3390/ijerph17207537

**Published:** 2020-10-16

**Authors:** Artur Wdowiak, Dorota Raczkiewicz, Paula Janczyk, Iwona Bojar, Marta Makara-Studzińska, Anita Wdowiak-Filip

**Affiliations:** 1 Diagnostic Techniques Unit, Medical University of Lublin, ul. Staszica 4/6, 20-081 Lublin, Poland; wdowiakartur@gmail.com; 2 Institute of Statistics and Demography, Collegium of Economic Analysis, SGH Warsaw School of Economics, ul. Madalińskiego 6/8, 02-513 Warszawa, Poland; dorota.bartosinska@sgh.waw.pl; 3 Nursing and Midwifery Institute, Faculty of Health Sciences, Jagiellonian University Medical College, ul. Kopernika 25, 31-501 Kraków, Poland; 4 Institute of Rural Health in Lublin, ul. Jaczewskiego 2, 20-090 Lublin, Poland; bojar.iwona@imw.lublin.pl; 5 Faculty of Health Sciences, Jagiellonian University Medical College, ul. Kopernika 25, 31-501 Kraków, Poland; marta.makara-studzinska@uj.edu.pl; 6 Department of Dermatology, Venerology and Pediatric Dermatology, Medical University of Lublin, ul. Radziwiłłowska 13, 20-080 Lublin, Poland; anita.wdowiak@gmail.com

**Keywords:** menstrual disorders, infertility, hypothalamic–pituitary–gonadal axis, cortisol, prolactin

## Abstract

One of the major problems of success in infertility treatment could depend on the understanding how the potential factors may affect the conception. The aim of this study was to evaluate present understanding of such factors or hormonal causes that may induce infertility. We studied the interactions between the two menstrual cycle hormones i.e., cortisol (COR) and prolactin (PRL), along with the ultrasonographic ovulation parameters in a group of *N* = 205 women with diagnosed infertility. The control group consisted of *N* = 100 women with confirmed fertility. In both groups, follicle-stimulating hormone (FSH), luteinizing hormone (LH), anti-Müllerian hormone (AMH), thyroid stimulating hormone (TSH), PRL, COR were examined on the third day of the cycle, and estradiol (E2), progesterone (P), and COR were examined during ovulation and 7-days afterwards. In the infertile group, higher levels of PRL and COR were observed than that of in the control group. Cortisol levels at all phases of the menstrual cycle and PRL negatively correlated with E2 secretion during and after ovulation, thus contributed to the attenuation of the ovulatory LH surge. Infertile women who conceived presented with higher levels of E2 during and after ovulation, higher P after ovulation, and thicker endometrium than that of the women who failed to conceive. In conclusion, elevated secretion of COR and PRL in infertile women impairs the menstrual cycle by decreasing the pre-ovulatory LH peak and E2 and postovulatory E2 levels that affect the endometrial growth, and consequently reduce the chances to conceive.

## 1. Introduction

Infertility is a global problem that is defined as the failure to achieve pregnancy within 12 months of regular sexual intercourse without the use of contraceptive methods. The World Health Organization (WHO) has noted that every fourth couple trying to have an offspring may experience difficulties in conceiving [1]. A considerable part of the causes of female infertility are caused by ovulation disorders. These disorders result, among other things, from the interaction between hormones of the hypothalamic–pituitary–gonadal (HPG) axis. In addition, hormones involved in stress exert an effect on the hypothalamic–pituitary–gonadal axis (HPG) [2]. In the course of the stress reaction, among other occurrences, there is an increased secretion of cortisol (COR) and prolactin (PRL) [3].

Hyperprolactinemia inhibits the pulsatile pattern of activity of the HPG and is considered one of the causes of infertility in males and females [4]. High concentrations of cortisol result in insensitivity of the pituitary gland to gonadotropin-releasing hormone and of the ovaries to luteinizing hormone (LH). The consequence is the limitation in the release of LH, follitropin (FSH), and estrogens; thus, when a prolongation of the follicular phase occurs, the menstrual cycle will become irregular and prolonged, and the probability of ovulation decreases.

The data in the literature are contradictory on whether the stress associated with inability to conceive is the main cause of ovulatory infertility or infertility itself generates emotional disorders leading to hormonal dysfunctions related to HPG dysfunction [3]. Although this challenge has yet to be addressed, it is known that proper gonadal function is involved in obtaining a good quality oocyte, and the proper level of sex hormones is directly related to achieving pregnancy. This idea has been confirmed in studies analyzing the chance for conceiving during frozen embryo transfer (FET) in the course of in vitro fertilization (IVF) [5,6,7]. In such cycles, estradiol and progesterone are supplemented to prepare the endometrium for FET, bypassing the HPG axis. In this case, obtaining proper levels of estradiol (E2) and progesterone (P) in the blood serum of women exerts a significant effect on achieving pregnancy [5,6,7].

Under physiological conditions, the daily production of P is 25 mg, which ensures serum P levels of 40–60 nmol/L (15–20 ng/mL). This secretion has a pulsatile pattern, and for approximately 30% of the time, the concentrations do not exceed 10 ng/mL; thus, 10 ng/mL has been adopted by the majority of researchers as the symptom of luteal phase failure. Supplementation with gestagens before frozen embryo transfer is aimed at the restoration of P concentrations to levels analogous to those occurring in the natural cycle [5,6]. Proper progesterone supplementation promotes becoming pregnant after frozen embryo transfer, and a low serum P level is associated with the risk of miscarriage. According to data from the literature, the mean progesterone value with FET was 11.3 ± 5.1 ng/mL, while a decrease in its level below 10.64 ng/mL resulted in an increased risk of therapy failure [7].

Estradiol secreted in the first phase of the cycle has multiple functions, including obtaining proper endometrial thickness for achieving pregnancy. Endometrial thickness (EMT) was found to be an independent factor affecting outcome; this finding implies that at a baseline live birth rate of 20%, an increase of 2 mm in EMT should result in an increase in the live birth rate by ≈1.6% [8]. Secretion of estradiol is conditioned by an adequate ovarian reserve, which is assessed by means of anti-Müllerian hormone (AMH), FSH, and LH. Secretion of estradiol is conditioned by an adequate ovarian reserve, which is assessed by means of AMH, FSH, and LH. Many scientific reports have analyzed ovarian reserve in the context of achieving pregnancy during infertility treatment [9,10]. Interactions between E2 and P are dependent on the ovarian reserve, and that, as well as a premature rise of progesterone during ovulation, can affect conception [11].

Clinically, in women suffering from thyroid disorders, it is known that frequent menstrual disorders, decreased fertility, and pregnancy failure occur [12]. There is evidence that thyroid hormones interact with FSH to exert a direct, stimulatory effect on the function of granular cells, including morphological differentiation, formation of the LH/HCG receptor, and induction of steroidogenesis enzymes (3 beta-hydroxysteroid dehydrogenase and aromatase) [13]. In addition, 1 3,5,3′-triiodothyronine induces estrogen receptor alpha mRNA expression, which suggests the hypothesis that the response at the level of the estrogen receptor is enhanced by T3 [14]. Thyroid function and prolactin are closely interrelated. An increased production of TRH, or less likely, a decreased dopamine turnover, could be responsible for the hypersecretion of TSH and prolactin in hypothyroidism.

Knowledge concerning the effect of the secretion of hormones on achieving pregnancy remains insufficient, despite considerable development of diagnostic tools and a large number of studies analyzing this problem. In relation to this evidence, a study was undertaken to determine whether there are any differences associated with the course of the sexual cycle in women diagnosed with infertility attempting to become pregnant naturally compared to those with confirmed fertility and how these differences affect the chance to achieve pregnancy. In the present study, attention was also paid to the levels of cortisol and its relationship with reproductive problems. In summary, the aim of this study was to assess the current understanding of the above-mentioned hormonal factors or causes that may induce infertility, due to the fact that the literature is poor in research on the topic presented and it has not yet been fully explained in the human model.

## 2. Materials and Methods

This was a prospective cohort study. The criterion for inclusion into the study group was the diagnosis of infertility according to the WHO classification [15]. The process of recruiting the study participants, both in the study group and in the control group, was carried out by two communication channels. The first one was to place printed advertisements about the study in local gynecological offices. The second was to place an ad with the same content online on a social networking site. Both advertisements were written in Polish, so the participants had to demonstrate their knowledge of language on a communicative level. The application to participate in the study was voluntary and supported by the interest shown by potential participants.

During the process of verification of study group participants, the interview method was used. Only women with a diagnosis of primary infertility (who were not previously pregnant) were qualified for the study, and according to the WHO definition of infertility [15], 1 year of regular efforts to achieve pregnancy has passed. Only women who first entered for infertility therapy, had not previously received hormone treatment, and their fallopian tubes were patent could be qualified as a participant. This type of infertility is classified as of unexplained etiology. The parameters of sperm of their partners met the WHO 2010 standards [16], which excluded male infertility. For the first 3 months after being diagnosed with infertility, women in the study group had their menstrual cycles monitored and naturally tried to become pregnant after the detection of an LH surge. In this study, the result of the first analyzed menstrual cycle is presented.

During the verification process of the control group participants, the interview method was also used. It was necessary for the woman to provide information about the previous pregnancy. At least one childbirth during the 2–4 years prior to the study was a condition for inclusion in the study. The criterion was established for a minimum of 2 years after pregnancy as a result from the need to exclude the likely effect of increased prolactin levels during breastfeeding on fertility disorders. The criterion of 4 years was adopted because it is the time during which the ovarian reserve should not be significantly reduced, therefore it could not have a potential impact on female fertility. In addition, the time to get pregnant could not exceed 1 year and the pregnancy could not be obtained by Assisted Reproductive Techniques (ART). The only acceptable form of contraception before the study was the use of a condom, as it has no effect on fertility. If a woman did not meet the conditions described above, she was excluded from the study. One menstrual cycle was monitored in the control group.

In both groups the criteria of exclusion from the study were the same, as follows: diagnosis of a chronic disease (metabolic, neurological, or cancerous); taking drugs or dietary supplements apart from folic acid; past psychiatric treatment or psychotherapy in medical history; ovulation disorders observed according to the WHO I criteria WHO (Groups I, II, III) [17]; body mass index (BMI) < 20 or >30 kg/m^2^, smoking, ethnicity other than Polish. To exclude hazardous, harmful, and dependent alcohol use WHO AUDIT (The Alcohol Use Disorders Identification Test) screen test was carried out [18].

The study was conducted in the OVEA gynecological-obstetric room in Lublin during the period from December 2018 to March 2019. The following protocol of the course of the study was established:

- Assessment of the course of the menstrual cycle:
  ○ *Determination of hormone levels*
    1. On the third day of the cycle-assessment of FSH, LH, AMH, TSH, PRL, COR;
    2. During ovulation, as determined using the ovulation test strips-assessment of E2, P, LH, COR;
    3. 7 days after ovulation-assessment of E2, P, COR;
    4. Determination of pregnancy: 2 weeks after ovulation-assessment of HCG.
  ○ *Ultrasound examination*
    1. During ovulation: assessment of the amount and size of dominant follicles (larger or equal to 17 mm), assessment of endometrial thickness;
    2. Seven days after ovulation: assessment of follicular rupture and assessment of endometrial thickness.
- Collection of sociodemographic data.

The estradiol values were then converted into the levels of estradiol per follicle. A dominant follicle was observed in the study. As the estradiol level depends on the number of dominant follicles, if two or more follicles ≥ 17 mm appeared, the estradiol value was divided into the number of follicles. All hormone levels were measured in serum obtained from morning blood samples (5 mL). The levels of selected hormones were assessed using one of two methods. The levers of COR, PRL, LH, FSH, E2, P, TSH were tested with electrochemiluminescence method from collected serum using the Cobas c6000 analyzer. HCG level was assessed with electrochemiluminescence method using Cobas e411 Rack Roche machine. AMH level was assessed using the method of chemiluminescence immunoassay using Beckman Coulter ACCESS machine.

Ultrasound examination was carried out in the same consultation room using an ALOKA ProSound SSD 3500 Ultrasound System.

Prior to the study, consent was obtained from the Bioethical Commission at the Medical University in Lublin, No. KE-0254/351/2018.

The obtained results were statistically analyzed using a method of comparing quantitative characteristics, Student’s *t*-test, for comparing independent samples. Considering the small number in the group of pregnant women, Mann–Whitney U tests were applied for quantitative comparisons between the groups with and without positive HCG test. The qualitative characteristics between the study and control groups were compared using tests. The correlation between the levels of prolactin and cortisol was investigated using the Pearson correlation coefficient r. Considering the large size of the samples for the study and control groups, according to the central limit theorem, it was assumed that the distributions of the parameters’ estimates are asymptotically normal. Values of *p* ≤ 0.05 were considered statistically significant.

## 3. Results

### 3.1. Characteristics of the Groups

During the process of recruitment, we obtained 139 applications for the control group. Among those, 132 met the required criteria. We randomly selected 100 participants among those eligible. One menstrual cycle was monitored, and all qualified participants took part in the study.

The willingness to participate in the study group was expressed by 224 women. During verification process, 208 women were qualified to participate. Three cycles were monitored in the study group. In this manuscript we reported only first from monitored cycles. Due to incomplete data three participants were excluded from the study.

The application for the study was voluntary. After providing full information on the form, course of the study, and possible risk, none of the participant refused to participate. A written consent was obtained from each participant.

The study included 205 women diagnosed with infertility according to the WHO criteria (study group, *N* = 205) and 100 women with confirmed fertility (control group, *N* = 100).

Table 1 presents their demographic characteristics. No differences between the study group and control group were observed in the following categories: age, level of education, type of occupation, time devoted to work, and family income. The women in the study group more rarely consumed alcohol than the women in the control group.

### 3.2. Comparison of the Menstrual Cycle Course between Study Group and Control Group

**Figure 1:**
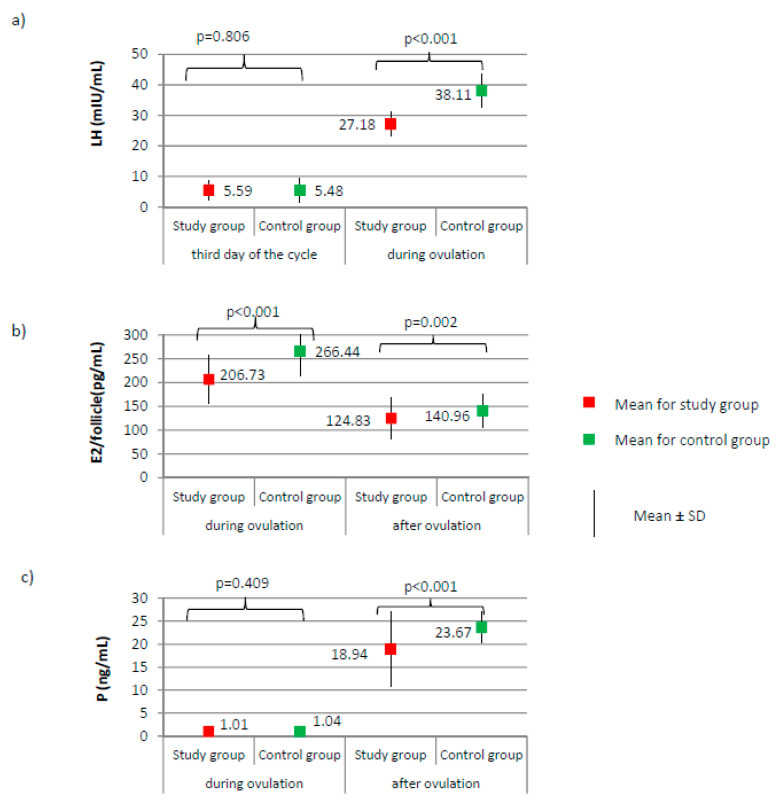
Comparison of LH (luteinizing hormone) (**a**), E2 (estradiol) (**b**), and P (progesterone) (**c**) between the study group (*N* = 205) and the control group (*N* = 100).

**Figure 2:**
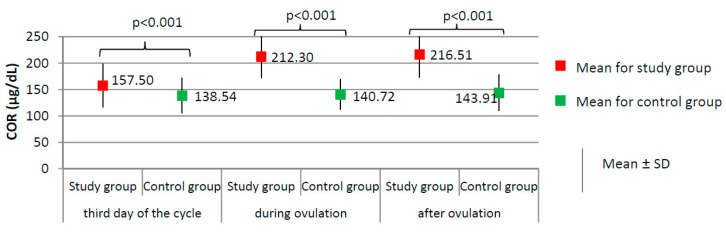
Comparison of COR (cortisol) between the study group (*N* = 205) and the control group (*N* = 100).

**Figure 3:**
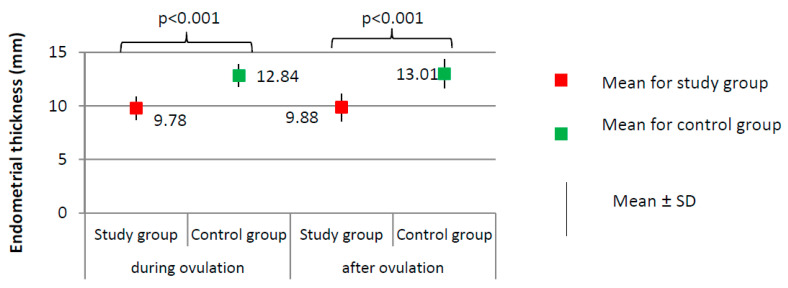
Comparison of endometrial thickness between the study group (*N* = 205) and the control group (*N* = 100).

Figure 1, Figure 2 and Figure 3 present the results of comparisons of menstrual cycle course between the study group (*N* = 205) and the control group (*N* = 100).

No significant differences between groups were found according to the levels of AMH and FSH on the third day of the cycle (Figure 2a,b, respectively). AMH level was on average 2.47 ± 0.72 for the study group versus 2.49 ± 1.00 ng/mL for the control group, *p* = 0.884. FSH level was on average 6.28 ± 1.87 for the study group versus 6.60 ± 2.00 mIU/mL for the control group, *p* = 0.172. The LH level did not significantly differ on day 3 of the cycle; however, during ovulation, it was significantly lower in the study group than it was in the control group (27.18 versus 38.11 mIU/mL; *p* < 0.001, Figure 1a). Compared to the control group, a significantly lower level of E2 was observed in the study group, both during ovulation (206.73 versus 266.44 pg/mL; *p* < 0.001) and after ovulation (124.83 versus 140.96 pg/mL; *p* < 0.001, Figure 1b). The examined groups did not differ according to the level of progesterone on the day of ovulation; however, a significant difference between groups was noted after ovulation (18.94 versus 23.67 ng/mL; *p* < 0.001, Figure 1c).

Women from the study group had significantly higher levels of cortisol at each phase of the menstrual cycle than did the women from the control group (*p* < 0.001, Figure 2), and higher prolactin levels on the third day of the cycle (24.75 ± 10.68 versus 17.78 ± 6.72 ng/dL; *p* < 0.001). No significant difference between the study group and the control group was found according to the levels of TSH on the third day of the cycle (1.76 ± 0.33 versus 1.71 ± 0.43 µIU/mL; *p* = 0.303).

Imaging tests did not show any significant differences with respect to the day of ovulation (12.41 ± 2.13 for study group versus 12.83 ± 1.54 for control group, *p* = 0.077) or the follicle size at ovulation (1.86 ± 0.20 for study group versus 1.90 ± 0.25 cm, *p* = 0.144). The percentage of women from the study group in whom the follicle ruptured (*N* = 189, 94.5%) did not significantly differ from the percentage in the control group (*N* = 96, 96.0%) (χ^2^ = 0.316; *p* = 0.574). Women from the study group had a significantly thinner endometrium than those in the control group, both during ovulation (9.78 versus 12.84 mm) and after ovulation (9.88 versus 13.01 mm; *p* < 0.001, Figure 3).

### 3.3. Correlations between Prolactin and Cortisol, and Menstrual Cycle Course in the Study Group and in the Control Group

Correlations between the levels of prolactin and cortisol and the levels of sex hormones, endometrial thickness, and follicle size were investigated separately in the study group (Table 2) and in the control group (Table 3).

Levels of prolactin on the third day of the cycle and the levels of cortisol after ovulation in women from the study group negatively correlated with the levels of LH during ovulation, estradiol levels during and after ovulation, progesterone levels after ovulation, endometrial thickness during and after ovulation, and follicle size (r < 0; *p* < 0.05) (Table 2). A negative correlation was observed between the cortisol level on the third day of the cycle and the level during ovulation, but the parameters over the course of the menstrual cycle were nearly the same as prolactin and cortisol levels after ovulation, with the exception of the follicle size in the study group. No correlations were noted between the levels of prolactin and cortisol and the levels of FSH and LH on the third day of the cycle or with progesterone during ovulation in the study group. This means that the higher the level of cortisol was on the third day of the cycle and during ovulation in women from the study group, the lower the levels of LH were, on average, during ovulation, estradiol levels were during and after ovulation, and progesterone levels were after ovulation; further, the endometrium was thinner in correlation with high cortisol.

In turn, in the control group, a smaller number of significant correlations was found between prolactin and cortisol levels and the menstrual cycle course compared to the study group (Table 3). The level of prolactin on the third day of ovulation positively correlated with the level of estradiol after ovulation (r = 0.227; *p* = 0.023), and it negatively correlated with follicle size in the control group (r = −0.221; *p* = 0.027). The level of cortisol on the third day of the cycle negatively correlated with the level of LH during ovulation (r = −0.369; *p* < 0.001) and the level of estradiol after ovulation (r = −0.520; *p* < 0.001). This means that the higher the level of cortisol was on the third day of the cycle in women from the control group, the lower the LH levels were, on average, during ovulation, and the lower estradiol levels were after ovulation. In the remaining cases, no significant correlations were observed in the control group.

### 3.4. The Menstrual Cycle Course in the Study Group—Comparison between Women with Positive HCG Test and Women with Negative HCG Test

**Figure 4:**
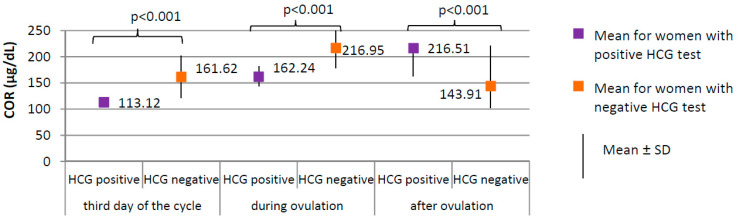
Comparison of COR (cortisol) between women with positive HCG test (*N* = 17) and women with negative HCG test (*N* = 188).

**Figure 5:**
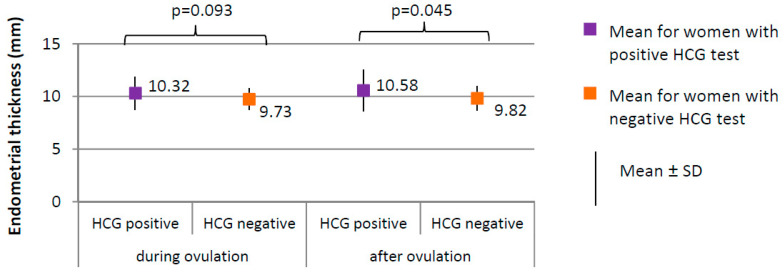
Comparison of endometrial thickness between women with positive HCG test (*N* = 17) and women with negative HCG test (*N* = 188).

**Figure 6:**
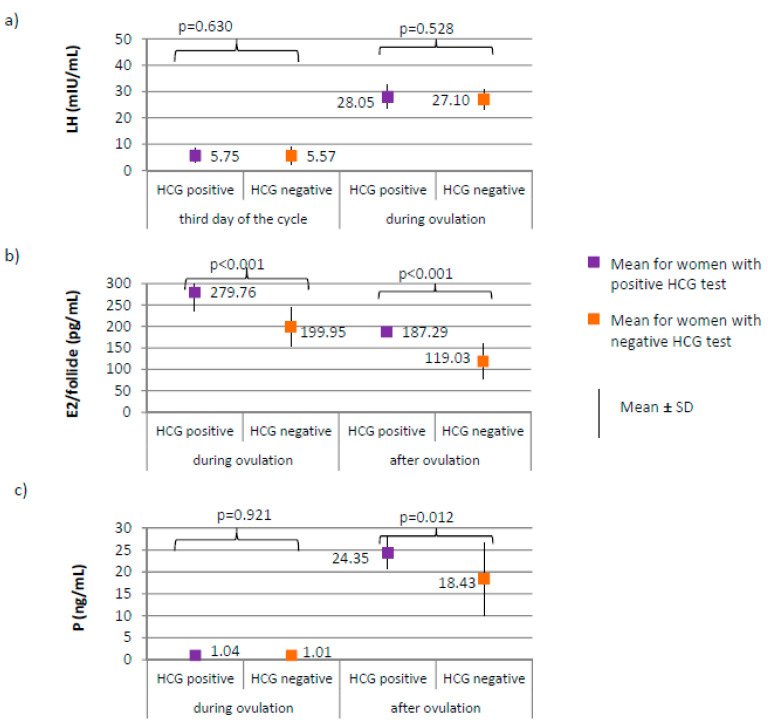
Comparison of LH (luteinizing hormone) (**a**), E2 (estradiol) (**b**), and P4 (progesterone) (**c**) women with positive HCG test (*N* = 17) and women with negative HCG test (*N* = 188).

Positive HCG test was achieved in 17 of 205 women from the study group (8.3%). Negative HCG test was achieved in 188 women from the study group (91.7%). Serum levels of the selected serum hormones and results of ultrasound examinations were compared between women from the study group with positive and negative HCG test (Figure 4, Figure 5 and Figure 6).

Women with positive HCG test, compared to those with negative HCG test, were characterized by a significantly lower level of cortisol at each phase of the cycle (*p* < 0.001; third day of the cycle 113.12 versus 161.62 µg/dL; during ovulation 162.24 versus 216.95 µg/dL; after ovulation 163.18 versus 221.46 µg/dL, respectively, Figure 4) and lower prolactin level on the third day of the cycle (18.31 ± 8.14 versus 25.35 ± 10.70 ng/mL, respectively; *p* = 0.009). No difference between groups was found in the levels of TSH on the third day of the cycle (1.59 ± 0.60 μIU/mL for women with positive HCG test versus 1.78 ± 0.29 μIU/mL for women with negative HCG test, *p* = 0.823).

The percentage of women with positive HCG test in whom the follicle ruptured (100.0%) did not significantly differ from women with negative HCG test in whom the follicle ruptured (183, i.e., 94.0%), (χ^2^ = 1.081; *p* = 0.298). Ovulation occurred on day 13, on average, in women with positive HCG test and on day 12 in women with negative HCG test (*p* = 0.064). The follicle size did not significantly differ between women with positive HCG test (1.87 ± 0.27 cm on average) and women with negative HCG test (1.86 ± 0.19 cm on average), (*p* = 0.775). No significant differences in endometrial thickness were observed during ovulation; however, the difference in endometrial thickness after ovulation was significant (*p* = 0.045; 10.58 mm) in women with positive HCG test versus 9.82 mm in women with negative HCG test (Figure 5).

No differences between women with positive and negative HCG test were found in the levels of AMH and FSH on the third day of the cycle. AMH level was on average 2.19 ± 0.52 for women with positive HCG test versus 2.49 ± 0.73 ng/mL for women with negative HCG test, *p* = 0.122. FSH level was on average 6.66 ± 1.97 for women with positive HCG test versus 6.24 ± 1.86 mIU/mL for women with negative HCG test, *p* = 0.348. LH on the third day, or during ovulation (Figure 6a) or progesterone level during ovulation (Figure 6c) did not significantly differ between women with positive and negative HCG tests. Women with positive HCG test, compared to those with negative HCG test, were characterized by a significantly higher estradiol level both during ovulation (*p* < 0.001; 279.76 versus 199.95 pg/mL) and after ovulation (187.29 versus 119.03 pg/mL), as well as a higher progesterone level after ovulation (*p* = 0.012; 24.35 versus 18.43 ng/mL) (Figure 6b,c).

## 4. Discussions

The conducted study is innovative and has no direct equivalent in the literature. It showed that one of the causes of infertility with unexplained etiology may be ovulation disorders caused by increased secretion of cortisol and prolactin. By recognizing this pathomechanism, it will be possible in the future to conduct targeted research on the etiology of this phenomenon and thus to implement appropriate therapy methods for women with this reproductive problem.

The results of the study demonstrate that in women with reproductive disorders, higher serum levels of cortisol and prolactin occur. Similar relationships were described by Csemiczky et al. in their study conducted on patients undergoing in vitro fertilization [19]. In the present study, women who became pregnant during treatment were characterized by significantly lower levels of COR and PRL than those who did not become pregnant. This is contrary to the results obtained by Csemiczky et al., Cesta et al., and Nouri et al. [19,20,21]. However, An et al., in their subsequent studies, confirmed the relationship between the levels of cortisol and prolactin and successfully becoming pregnant [22]. In addition, Nepomnaschy P. A. et al. showed that an increased cortisol level contributes to the occurrence of miscarriages during the initial period of development of pregnancy, which suggests that this phenomenon also concerns implantation disorders at a very early stage, even just after the embryo enters the blastocyst stage, which, in turn, may explain the results of higher cortisol values obtained in the group where HCG test was negative [23].

Cortisol is a steroid hormone produced from cholesterol in the adrenal cortex and is the main but not the only hormone involved in a stress response. Its steroidal structure enables easy penetration through the phospholipid membranes of cells, which in turn determines the rapid response of the cell to the action of this hormone. Psychological stress is a change occurring in psychological regulatory mechanisms and activities under the influence of various types of difficult life situations. It is not known whether the mere failure to obtain offspring contributes to stressful reactions and excessive secretion of COR, or whether increased secretion of this hormone has another cause. Moreover, in addition to Cushing’s syndrome, hypercortisolism is found in 2–5% of individuals with obesity, metabolic unbalanced type 2 diabetes, and hypertension [24].

In many studies, proper levels of AMH and FSH are predictive factors for successfully becoming pregnant. AMH belongs to the transforming growth factor beta family (TGFβ). During fetal life, its main known role is to induce the disappearance of Müller’s ducts in male fetuses. On the other hand, in women, AMH begins to play an important role only during adolescence and later in the reproductive period. AMH participates in the regulation of folliculogenesis, inhibiting the process of recruitment of embryonic follicles by reducing the influence of FSH on the growth of preantral and antral follicles. The results of experimental studies indicate that AMH in the paracrine mechanism inhibits embryonic vesicle maturation and estradiol production by inhibiting aromatase activity in granular cells [25,26]. The importance of AMH in human reproduction is confirmed by research of Hussain et al., Lehmann et al., Reichmanm et al., and Wdowiak et al., which indicate that the chance for achieving pregnancy grows together with an increase in the level of AMH in the case of treatment by the in vitro fertilization method. The abovementioned studies did not unequivocally show whether FSH or AMH has a greater predictive value in achieving pregnancy [10,27,28,29]. In this study, women with very high AMH (polycystic ovarian syndrome) and those with lowered levels of this hormone (premature menopause) were excluded. Thanks to the homogeneity of the group in terms of AMH levels introduced in this way, it can be concluded that in the presented study, the secretion of this hormone was not associated with a chance of obtaining a positive HCG test result. This is consistent with Seckin et al., Casadei et al., and Pacheco et al. studies. Those researchers indicated that in the case of infertility of unexplained cause, AMH may not be applicable in predicting the possibility of achieving pregnancy [30,31,32].

In this research, no significant differences in the LH level were observed between the group of women who became pregnant and the group who did not. Similar observations were presented in the studies by Wdowiak et al. and Ramachandran et al. [9,33].

It was confirmed that the women who, during infertility treatment, are characterized by having higher E2 levels during the whole cycle and higher P levels during the second phase have better chances for becoming pregnant. The same result was obtained by Csemiczky et al. [19]. Similar relationships with respect to the levels of estrogen but concerning in vitro fertilization were also observed. In these investigations, at a higher E2 level, the embryos obtained a better quality and dynamics of development [9,33,34,35].

In a study by Murto et al., a level of P above 32 nmol/L (10.06 ng/mL) was the predictive factor for achieving pregnancy, but also predictive was a TSH level below 2.5 mIU/L and an AMH level over 10 pmol/L (1.4 ng/mL) [36]. In the present study, neither the TSH nor AMH levels caused differences in conceiving, probably because these results remained within the reported ranges. However, the P values also remained beyond the range reported by the researchers, and despite this, a significant difference was demonstrated between the results obtained in the examined women. Those in whom the P level remained at approximately 58 nmol/L (18 ng/mL), on average, did not become pregnant during treatment.

According to Diedrich K. et al., pregnancy begins with successful fertilization and implantation, which greatly depends on endometrial receptivity [37]. According to Simón C. et al., several factors, such as estradiol, progesterone, cytokines, and neuropeptides, as well as glucocorticoids, regulate this complex effect [38,39]. Endometrial thickness was reported as a predictive value for the achieving pregnancy by Dinelli et al. when it oscillates between 10 and 11 mm [40]. A similar result was obtained in the present study. Women whose endometrium after ovulation was below 10 mm did not become pregnant. In a study carried out by Gao et al., the results of a meta-analysis of studies of more than 88,000 cycles also confirmed that women characterized by having a thinner endometrium achieved pregnancy more rarely than women with a thicker endometrium [41].

One of the pathomechanisms leading to decreased fertility considered by Prasad S. et al. is a decrease in the secretion of E2 as a result of intensification of the stress-related production of COR [42]. The results of the present study confirm this notion (the occurrence of a negative correlation between cortisol secretion and the levels of E2 and P). According to Prasad S. et al., this phenomenon is responsible for a reduction in the quality of the egg cells produced and, consequently, a decrease in the percentage of achieved pregnancies.

Higher cortisol levels were observed on the third day of the cycle and during ovulation in women from the study group, and they exhibited lower average LH levels during ovulation, estradiol levels during and after ovulation, progesterone levels after ovulation, and thinner endometrium. The LH level had no effect on the achievement of pregnancy; however, the levels of the remaining hormones exerted an effect.

The results of the study should be interpreted with caution, taking into consideration several limitations. Considering the relatively small number of examined women who became pregnant during treatment, the application of generalized conclusions is limited. In addition, precise hours of collection of cortisol and collection of other hormones were not considered; therefore, it was impossible to account for daily variability, which may result in the deterioration of the strength of the study. It should be kept in mind that this study focused exclusively on a specifically selected group of women with fertility disorders of undefined etiology and it may not be applicable to other types. However, despite the abovementioned limitations, the present study also has its strengths. The results obtained and the correlations confirmed concerning cortisol and prolactin as predictive factors for the achievement of pregnancy would provide women treated for infertility with better diagnostics due to the prospective characteristics and easy measurement of specific parameters. Assessment is recommended of the levels of cortisol and prolactin due to their effect on the levels of estradiol and progesterone and endothelial thickness after ovulation, which is directly related to the possibility of becoming pregnant during infertility treatment.

## 5. Conclusions

1. Women have better chances to become pregnant if they have a lower level of cortisol during the course of the menstrual cycle, a lower level of prolactin, a higher level of estradiol in the whole cycle, and a higher level of progesterone after ovulation, as well as thicker endometrium before and after ovulation.

2. Higher levels of cortisol and prolactin exert a negative effect on estradiol secretion during ovulation and contribute to the attenuation of the ovulatory LH surge.

## Figures and Tables

**Table 1:** Demographic characteristics of the examined groups.

Demographic Data	Study Group (*N* = 205)	Control Group (*N* = 100)	χ^2^ or t, *p*
Age—years M ± SD (min–max)	26.7 ± 1.9 (23–30)	26.8 ± 1.8 (23–30)	t = 2.266 *p* = 0.790
Place of residence *n* (%)			
Urban	90 (45%)	45 (45%)	χ^2^ = 0.000
Rural	115 (55%)	55 (55%)	*p* = 1.000
Education *n* (%)			
Higher	49 (24%)	21 (21%)	χ^2^ = 2.790
Secondary school	78 (38%)	31 (31%)	*p* = 0.248
Primary school	78 (38%)	48 (48%)	
Type of occupation *n* (%)			
Physical	84 (41%)	47 (47%)	χ^2^ = 1.029
Intellectual	82 (40%)	35 (35%)	*p* = 0.598
Mixed	31 (19%)	18 (18%)	
Number of hours of work weekly *n* (%)			
34–39	135 (66.5%)	68 (68%)	χ^2^ = 1.951
40–44	33 (16%)	11 (11%)	*p* = 0.583
45–49	17 (8%)	8 (8%)	
50–55	20 (9.5%)	13 (13%)	
Family income *n* (%)			
High	46 (22.5%)	30 (30%)	χ^2^ = 3.434
Mediocre	111 (54%)	43 (43%)	*p* = 0.180
Low	48 (23.5%)	27 (27%)	
Alcohol consumption *n* (%)			
No	191 (94%)	64 (64%)	χ^2^ = 44.643
Yes	14 (6%)	36 (36%)	*p* < 0.001
Age of the spouse—years (exclusively study group) M ± SD (min–max)	33.0 ± 5.7 (23–49)	-	-

**Table 2:** Correlations between prolactin (PRL) and cortisol (COR) levels and the menstrual cycle course in women from the study group (*N* = 205). Significant correlations are in bold. Pearson’s correlation coefficient was applied.

Hormones	Menstrual Cycle Phase	PRL (ng/mL)		COR (µg/dL)					
		Third Day of the Cycle		Third Day of the Cycle		Ovulation		After Ovulation	
		r	*p*	r	*p*	r	*p*	r	*p*
FSH (mIU/mL)	Third day of the cycle	0.096	0.176	0.000	0.997	0.052	0.468	0.015	0.832
LH (mIU/mL)	Third day of the cycle	0.016	0.821	−0.011	0.879	0.015	0.831	0.006	0.937
	Ovulation	−**0.187**	**0.008**	−**0.224**	**0.001**	−**0.165**	**0.020**	−**0.226**	**0.001**
E2/follicle (pg/mL)	Ovulation	−**0.569**	**<0.001**	−**0.857**	**<0.001**	−**0.820**	**<0.001**	−**0.882**	**<0.001**
	After ovulation	−**0.670**	**<0.001**	−**0.890**	**<0.001**	−**0.884**	**<0.001**	−**0.899**	**<0.001**
P (ng/mL)	Ovulation	−0.001	0.996	−0.033	0.641	−0.008	0.910	−0.035	0.620
	After ovulation	−**0.705**	**<0.001**	−**0.840**	**<0.001**	−**0.765**	**<0.001**	−**0.823**	**<0.001**
Endometrial thickness (mm)	Ovulation	−**0.259**	**<0.001**	−**0.246**	**<0.001**	−**0.333**	**<0.001**	−**0.321**	**<0.001**
	After ovulation	−**0.268**	**<0.001**	−**0.269**	**<0.001**	−**0.357**	**<0.001**	−**0.349**	**<0.001**
Follicle size (cm)	Ovulation	−**0.150**	**0.034**	−**0.130**	**0.066**	−**0.108**	**0.128**	−**0.152**	**0.032**

Legend: follicle-stimulating hormone (FSH), luteinizing hormone (LH), estradiol (E2), progesterone (P), prolactin (PRL), cortisol (COR).

**Table 3:** Correlations between prolactin (PRL) and cortisol (COR) levels and the menstrual cycle course in women from the control group *(N* = 100). Significant correlations are in bold. Pearson’s correlation coefficient was applied.

Hormones	Menstrual Cycle Phase	PRL (ng/mL)		COR (µg/dL)					
		Third Day of the Cycle		Third Day of the Cycle		Ovulation		After Ovulation	
		r	*p*	r	*p*	r	*p*	r	*p*
FSH (mIU/mL)	Third day of the cycle	0.175	0.082	−0.071	0.481	−0.065	0.524	−0.053	0.600
LH (mIU/mL)	Third day of the cycle	−0.008	0.941	0.040	0.691	0.059	0.557	0.019	0.852
	Ovulation	−0.017	0.871	−**0.369**	**<0.001**	−0.031	0.763	−0.046	0.653
E2/follicle (pg/mL)	Ovulation	−0.117	0.245	−0.125	0.216	0.019	0.851	−0.041	0.683
	After ovulation	**0.227**	**0.023**	−**0.520**	**<0.001**	−0.118	0.241	−0.141	0.161
P (ng/mL)	Ovulation	−0.018	0.860	0.027	0.792	−0.034	0.737	−0.046	0.652
	After ovulation	−0.024	0.814	−0.109	0.282	0.004	0.969	−0.003	0.974
Endometrial thickness (mm)	Ovulation	0.159	0.115	0.164	0.104	−0.045	0.659	−0.015	0.882
	After ovulation	0.177	0.078	0.093	0.358	−0.066	0.512	−0.033	0.746
Follicle size (cm)	Ovulation	−**0.221**	**0.027**	0.193	0.054	−0.033	0.744	−0.048	0.636

Legend: follicle-stimulating hormone (FSH), luteinizing hormone (LH), estradiol (E2), progesterone (P), prolactin (PRL), cortisol (COR).
